# Widespread Reductions of Spontaneous Neurophysiological Activity in Leber’s Disease—An Application of EEG Source Current Density Reconstruction

**DOI:** 10.3390/brainsci10090622

**Published:** 2020-09-08

**Authors:** Kamil Jonak

**Affiliations:** Department of Clinical Neuropsychiatry, Medical University of Lublin, 20-439 Lublin, Poland; kamil.jonak@umlub.pl

**Keywords:** EEG, LHON, alpha band, gamma band, current-source density

## Abstract

Leber’s hereditary optic neuropathy (LHON) is a rare, maternally inherited genetic disease caused by a mutation of mitochondrial DNA. Classical descriptions have highlighted structural abnormalities in various parts of patients’ optic tracts; however, current studies have proved that changes also affect many cortical and subcortical structures, not only these belonging to the visual system. This study aimed at improving our understanding of neurophysiological impairments in LHON. First of all, we wanted to know if there were any differences between the health control and LHON subjects in the whole-brain source electroencephalography (EEG) analysis. Second, we wanted to investigate the associations between the observed results and some selected aspects of Leber’s disease’s clinical picture. To meet these goals, 20 LHON patients and 20 age-matched healthy control (HC) subjects were examined. To investigate the electrophysiological differences between the HC and LHON groups, a quantitative analysis of the whole-brain current source density was performed. The signal analysis method was based on scalp EEG data and an inverse solution method called low-resolution brain electromagnetic tomography (eLORETA). In comparison with the healthy subjects, LHON participants showed significantly decreased neuronal activity in the alpha and gamma bands; more specifically, in the alpha band, the decrease was mainly found in the occipital lobes and secondary visual cortex, whereas, in the gamma band, the reduced activity occurred in multiple cortical areas. Additionally, a correlation was found between the alpha band activity of the right secondary visual cortex and the averaged thickness of the right retinal nerve fiber layer in the LHON participants. Our study suggests that LHON is associated with widespread cortical de-activation, rather than simply abnormalities of structures constituting the visual system.

## 1. Introduction

Leber’s hereditary optic neuropathy (LHON) is a maternally inherited genetic disease caused by a mutation of mitochondrial DNA (mtDNA) that causes central, bilateral, and progressive visual loss due to optic nerve atrophy, especially in young adult men [1,2]. Three disease-causing mutations that affect complex I subunits of the mitochondrial respiratory are responsible for 90% of the cases (MTND1: m.3460G > A, MTND4: m.11778G > A, and MTND6: m.14484T > C) [3]. LHON’s key pathological characteristics include cell degeneration in the retinal ganglion and the optic nerve axonal detachment. In most cases, the early stage of the disease is characterized by the gradual optic nerve atrophy caused by the loss of smaller caliber fibers of the papillomacular bundle [4,5].

The classic 19th-century picture of LHON is used to describe mainly the ophthalmic dysfunctions; however, the development of a wide range of magnetic resonance imaging techniques (MRI), such as diffusion (DTI—diffusion tensor imaging) or functional (fMRI—functional magnetic resonance imaging) protocols, has expanded the current knowledge about the impact of mtDNA diseases on the human central nervous system (CNS) [6,7]. Researchers reported that in LHON participants, CNS abnormalities occurred in different parts of the visual tract, such as the optic chiasm or optic radiations [8,9]. However, recent anatomical studies have also shown some abnormalities beyond the optic tract, i.e., an enlarged ventricular system or visual cortex thickening [8,10]. These findings clearly suggest that pathological brain changes caused by the disease are not only limited to the optic nerve area. Apart from anatomical MRI studies, some surveys also analyzed LHON participants’ brain activity in a resting state (RS). The results of these fMRI studies showed the occurrence of functional abnormalities among LHON participants, especially decreased activity values in the right lateral occipital cortex and right temporal occipital fusiform gyrus [11,12]. These abnormalities were also significantly correlated with retinal damage and disease duration.

Electroencephalography (EEG) is another functional neuroimaging method that allows for the assessment of electrical potentials of the brain [13,14]. In comparison to fMRI, the application of EEG enables the accurate observation of the neural processes occurring in the brain cortex with very high temporal precision [15]. Previous studies that examined electrophysiological changes in LHON subjects most often investigated various aspects of the visual evoked potentials (VEP) response recorded in the patients’ occipital cortex [16]. The results of studies with VEP analysis also showed a decreased N95 component in LHON participants [17], and a low amplitude could be seen in the acute stage of LHON, together with an abnormal potential latency [18]. Nevertheless, apart from the research focused on visual nerve electrophysiological properties, there is a lack of group studies that describe global EEG abnormalities in the LHON group, but there are some studies devoted to the assessment of epileptic changes or the myoclonus mechanism in the EEG LHON patterns [19,20].

The main aim of the EEG signal analysis in clinical and research settings is the feature extraction from the signal. For that purpose, researchers are constantly developing new methods of signal analysis that could help to understand such a complex system as the human brain [21,22]. Recent signal analysis frameworks based on matching learning algorithms or the recurrence quantification analysis were found to be very useful tools for EEG signal analysis [23,24,25] or statistical inquiry [26,27]. Nevertheless, besides the extraction of features from the EEG signal, an equally important issue involves cleaning the signal of erroneous information and artifacts. The EEG signal can be affected by the occurrence of the volume conduction error (complex effects of measuring electrical potentials at a distance from their source generators), movement artifacts, or electrical noise [15]. The volume conduction effect is connected with the propagation of a current flow caused by synchronized post-synaptic potentials of pyramidal neurons in the brain. Using Poisson’s equations, it can be said that the electrical potential differences between electrodes are positioned on distinct scalp locations. Additionally, these potentials do not propagate homogenously since the electrical flow is attenuated by the neurophysiological processes in the brain and the resistivity of the skull; hence, the correct identification of signal source in the brain is difficult to achieve [28]. To overcome these effects, a method based on a limited number of equivalent dipoles has been applied to the EEG signal analysis. Recent development methods based on distributed source localization, such as low-resolution electromagnetic tomography (eLORETA) allow researchers to analyze EEG signals without volume conduction errors [29].

This study aimed to investigate whole-brain EEG changes in a group of LHON participants in comparison to the healthy controls (HC). Previous studies that examined the structural and functional abnormalities in LHON patients showed that pathological characteristics are not limited to only visual pathway areas; hence, it is important to expand our knowledge about probable global electrophysiological abnormalities that are present in LHON participants. For this purpose, and to avoid a volume conduction problem in the EEG analysis, the electrophysiological signal sources were reconstructed based on the recordings of a 64-channel EEG. The additional goal was to establish hypothetical associations between the values of the current source density, which significantly differentiated the groups, and the LHON clinical parameters, such as illness duration or retinal nerve fiber layer (RNFL) measures.

## 2. Methods

### 2.1. Participants

First, we found 70 participants in the national database that had received an LHON diagnosis. Second, for further processing, we discarded the participants with a different mtDNA mutation than 11778G > A, which was confirmed by genetic tests, and at this point, only 43 participants met this criterion. Third, we excluded from the analysis the participants who did not meet other inclusion criteria, such as right-handedness; lack of pathologies within the cerebrovascular system; no family history of severe neuropsychiatric disorders; not suffering from diabetes, hypertension, or any other neurodegenerative diseases; no history of chronic drugs or alcohol consumption; non-smoker; aged over 18; minimum of 10 years of education; no previous idebenone treatment (as well as during our tests). Only 30 participants were invited to individual interviews with the medical and research staff. Six participants did not agree to participate in the experiment, and the medical histories of four of them from the database were found to be incomplete (i.e., some of them had a history of severe substance abuse and some were left-handed). Finally, after the whole selection process, only 20 patients were found to meet the criteria and took part in the experiment. Two of our participants were related and had a family history of LHON. Additionally, none of the patients reported receiving any additional treatment for ocular disease. The HC group was matched by age and gender and was recruited from the local community. This research was approved by the local medical ethics committee of the Medical University of Lublin (KE-0254/23/2017) and was carried out in compliance with the national legislation and the Declaration of Helsinki [10]; all of the participants signed an informed consent form.

### 2.2. EEG Recording

First, 12 min of resting-state EEG (eyes closed) data were recorded from all of the participants (20 LHON and 20 HC). EEG examinations were done in a well-lit and quiet room with the use of a 64-channel HydroCel Geodesic Sensor Net (Electrical Geodesics Incorporated, Eugene, OR, USA) with Ag-AgCl electrodes. During the data recording, the sampling rate was kept at 1 kHz with the application of a vertex reference using the NetStation software package (Electrical Geodesics Incorporated, Eugene, OR, USA). Impedances of the electrodes were maintained below 65 kΩ. Additionally, during the recording, a band-pass filter (0.5 to 70 Hz) and an active notch filter were used (50 Hz). Visual inspection of the spline interpolation of bad channels was made. After having been recorded, the NetStation software (Electrical Geodesics Incorporated, Eugene, OR, USA) was used for the data conversion to the ASCII format.

Second, ASCII files with the EEG recordings were imported to EEGLAB v.13.5.4b1 (http://sccn.ucsd.edu/eeglab/index.html; [30]), which is an open-source toolbox for MATLAB (Mathworks, Inc., Natick, MA, USA). Then, the EEG signals were re-referenced to the common average reference offline and filtered with a bandpass Hamming window 0.5–45 Hz filter. After the filtering procedure, each EEG data set was segmented into 150 epochs, with a duration of 4096 samples (approximately 4 s) each. After the segmentation, each epoch was visually inspected by a certified clinical neurophysiologist, who removed bad epochs containing artifacts (i.e., head or muscle movements, electrode cable movements, or jaw clenching) from the analysis. Finally, for each participant, 135 visual-artifact-free epochs were selected [14].

### 2.3. EEG Source Localization and Functional Connectivity Analysis

**Figure 1:**
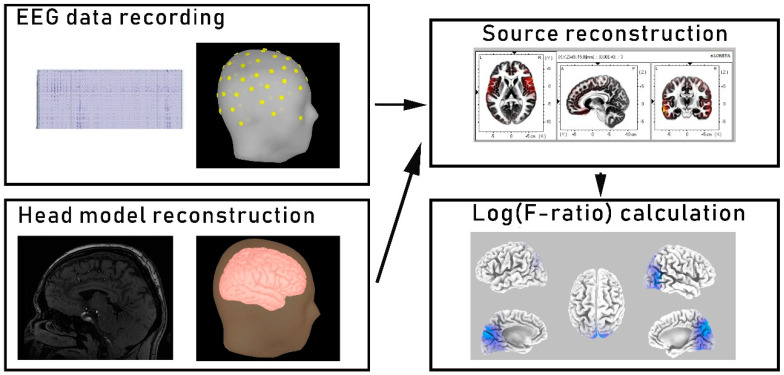
Source reconstruction analysis flowchart. In the first step, the electroencephalogram (EEG) signals were recorded, filtered, and segmented. Second, the head model was reconstructed using the MNI152 scalp template. Third, sources were reconstructed by applying the low-resolution brain electromagnetic tomography (eLORETA) algorithm for every participant and in every frequency band. Finally, the differences in source localization (in each frequency band) between the two groups were evaluated using voxel-by-voxel independent sample F-ratio tests.

For the source localization estimation, the eLORETA software was used [31], where Figure 1 presents the analysis flowchart. The eLORETA algorithm allows for the analysis of EEG signals with zero localization error under noise-free conditions. These properties are ensured primarily by the analysis being done on a discrete three-dimensionally distributed linear inverse solution of the EEG signal; hence, the images obtained represent the current density and present the point of exact source localization [29,32]. Nevertheless, the spatial resolution of the results was still low due to the highly correlated neuronal sources that underlie the EEG signals, but the volume conduction errors were removed from the analysis. The lack of bias in the EEG estimation is another important feature of the algorithm. Additionally, to test point sources, the linearity and superposition of the localization errors can determine the localization properties of all 3D inverse solutions. Thus, it can be said that if the distribution has no localization error to the point sources, located anywhere in the brain, then any arbitrary 3D distribution can be properly localized in the tomography (excluding distribution maps with a low spatial resolution). Moreover, detailed descriptions of the eLORETA algorithm were presented in different publications [33]. In this study, the intracerebral volume was restricted and split into 6239 voxels with a spatial resolution of 5 × 5 × 5 mm and the MNI152 scalp template was used for the standard electrode positions [34]. Finally, estimated current densities were presented on the eLORETA images as the exact magnitude of the electric activity of each voxel in the Montreal Neurological Institute (MNI) space. Additionally, the current source density analysis was performed for five frequency bands: delta (1–4 Hz), theta (4–8 Hz), alpha (4–12 Hz), beta (13–30 Hz), and gamma (30–45 Hz) [32].

### 2.4. Optical Coherence Tomography (OCT) Acquisition

Each subject from the LHON group was scanned at least three times, where only the best quality images were used for the analysis. The RNFL examination was performed using OCT (Revo NX 130, Optopol, Poland). A trained technician removed images from the analysis set in which the signal strength was lower than 6 and images that contained blinking artifacts. The RNFL scan protocol used a preset diameter of 3.45 mm and was centered on the optic nerve disk. The averaged RNFL thickness was ranked using the OCT computerized algorithm against a normal percentile. We used four different categories of the distribution scale that were based on age-matched controls: markedly below normal (<1% percentile), below normal (<5th percentile), normal (5–95th percentile), and supra-normal (>95th percentile). During the scanning, the internal fixation was always used and pupil dilatation was induced in all the subjects.

### 2.5. Statistical Analysis

The differences in source localization (in each frequency band) between the two groups were evaluated using voxel-by-voxel independent sample F-ratio tests, which were derived from the power of the log-transformed current density. A nonparametric permutation/randomization method (i.e., based on the Fisher permutation method, with the threshold set at the 5% probability level) defined the acquired statistical three-dimensional images, as represented by cortical voxels showing significant differences. The randomization strategy defined the critical probability threshold values for the observed t-values along with the correction for multiple comparisons across all frequencies in all voxels. Additionally, due to the evaluation of the empirical probability distribution of the “maximum statistics” in the null hypothesis, the permutation and randomization tests were found to be successful at regulating type I errors in recent studies [28]. For this analysis, a total of 15,000 permutations were used for each randomization test to evaluate the critical likelihood threshold values for the log(F-ratio) values with a correction for multiple comparisons across all voxels, without relying on the Gaussianity. Several previous studies have confirmed the suitability of the use of the SnPM procedure applied to eLORETA images [22,29]. Additionally, the eLORETA non-parametric randomization approach based on the “full statistics” was implemented for the correction of multiple comparisons. After establishing a set of source signals that significantly differentiated the groups, the results were correlated with selected clinical (e.g., duration of illness) and ophthalmologic characteristics (e.g., RNFL) using Pearson r-tests and FDR (false discovery rate) corrections. The correlations were computed only in the LHON sample and performed in the STATISTICA 13 package (StatSoft Inc., Tulsa, OK, USA).

## 3. Results

### 3.1. Participants

The demographic and clinical data of both studied groups are presented in Table 1. The selected groups did not differ significantly in terms of sex (LHON = 90% male; HC = 90% male), age (LHON = 34.8 years; HC = 32.35 years), and time of education (LHON = 15.2 years; HC = 15.8 years). The illness duration of the LHON participants was about 11 years and the RNFL average thickness was left: 62.105 µm and right: 61.765 µm.

### 3.2. Current Source Density

**Figure 2:**
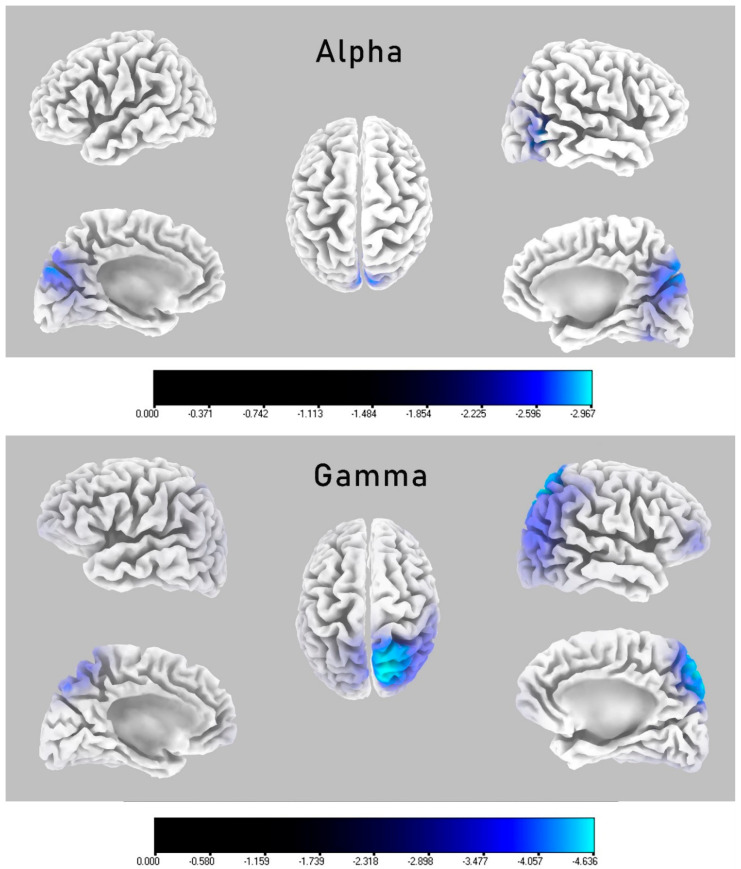
eLORETA spatial maps of voxels that were significantly differentiating groups with respect to current source density distribution on a cortical surfaces. Differences were labelled with different shades of blue due to decreased oscillations in the LHON group, with brighter spots of peak-to-max difference voxels according to log F-ratio values. Each segment displays lateral and medial projections of the left and right hemisphere and the top view. Distribution of alpha frequency band (upper map) shows that LHON participants had significantly decreased current source density values in occipital and parietal lobes, with the voxels of peak difference in the right middle occipital gyrus (*t*_max_ = −3.86) and the secondary visual cortex (*t*_max_ = −3.59). The lower map presents the distribution in the gamma frequency band, where LHON participants had significantly decreased current source density values in multiple brain areas, such as the parietal, occipital, and frontal lobes. The voxels o peak difference was placed in the right superior parietal lobule (*t*_max_ = −4.11), right precuneus (*t*_max_ = −4.1), right angular gyrus (*t*_max_ = −4.03), and right inferior parietal lobule (*t*_max_ = −4.01). The low log(F-ratio) significance threshold for the gamma frequency band was log(F-ratio) = −2.21 with *p* < 0.05.

Comparative analysis of the groups in terms of the resting-state current source density results, performed using the eLORETA statistical software application, showed significant differences in the alpha and gamma frequency bands (Table 2). Figure 2 provides maps and estimates of the spatial distribution of the voxels, which significantly differentiated the groups in both frequencies regarding the current source density results. In both the alpha and gamma frequency bands, the neuronal activity in the LHON group was significantly reduced compared to the controls, with a statistical significance threshold for the log(F-ratio) of −2.21 (*p* < 0.05). In the alpha band, the LHON patients had a significantly lower current source density bilaterally in the occipital lobes compared with the HCs, with the voxel of peak difference placed in the middle occipital gyrus (*t*_max_ = −3.86, *p* < 0.001; Brodmann area (BA) 37) and secondary visual cortex (*t*_max_ = −3.59, *p* = 0.001; BA 19). In the gamma frequency, significant differences occurred in multiple brain areas, such as the parietal, occipital, and frontal lobes. Nevertheless, the voxel of peak difference was placed bilaterally in the superior parietal lobule (*t*_max_ = −4.11, *p* < 0.001; BA 7), right precuneus (*t*_max_ = −4.1, *p* < 0.001; BA 7), right angular gyrus (*t*_max_ = −4.03, *p* < 0.001; BA 39), and right inferior parietal lobule (*t*_max_ = −4.01, *p* < 0.001; BA 40).

### 3.3. Associations between the Clinical Features of LHON Patients and Current Source Density Results

**Figure 3:**
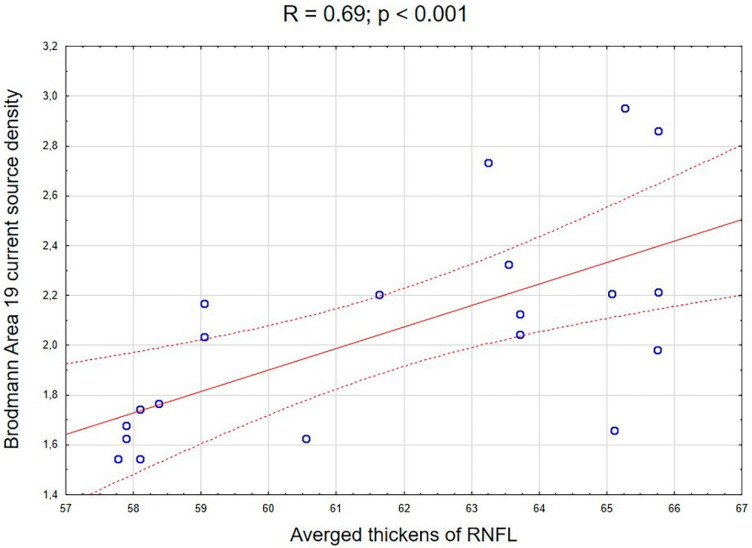
A correlation scatterplot showing the associations between the averaged thickness of the right retinal nerve fiber layer and the right hemisphere BA 19 source signal in the alpha frequency band in the LHON group.

To identify the potential associations between the abnormal current source density in the LHON group and the clinical picture of the disease, signal sources were mapped onto selected regions of interests (ROIs) corresponding to specific Brodmann areas (i.e., BA 37 in the alpha frequency band). Chosen ROIs were generated using the eLORETA software. Among all the included variables, only the correlation between the right hemisphere BA 19 source signal in the alpha band and the averaged thickness of the right retinal nerve fiber layer survived the FDR correction for the threshold of statistical significance: r = 0.69, *p* < 0.001. Figure 3 shows the correlation scatterplot.

## 4. Discussion

The main goal of this study was to investigate the whole-brain EEG changes in a group of patients with Leber’s disease by comparing them with demographically matched healthy controls. For this purpose, groups were analyzed regarding differences in the distribution of the current source density spreading throughout the cortical areas. Furthermore, the observed neurophysiological abnormalities were significantly associated with the features of the clinical disease picture. We observed that in comparison to the controls, LHON patients showed a significantly decreased resting-state current source density in the alpha and gamma frequencies. In the alpha band, the main differentiating areas were located in the middle occipital gyrus and secondary visual cortex. The main areas that differentiated the groups in the gamma frequency occurred bilaterally in the superior parietal lobule, right inferior parietal lobule, angular gyrus, and right precuneus, although significant differences were also observed in other cortical areas, including the frontal, parietal, temporal, and occipital lobes. Furthermore, the right hemisphere BA 19 source signal in the alpha band was significantly correlated with the average thickness of the right RNFL in the LHON participants.

Deviated EEG activity in the alpha frequency is one of the most frequently reported aberrations of the EEG signal in a wide range of diseases [35,36,37]. Alpha oscillations predominantly originate from the occipital lobe during wakeful relaxation with closed eyes in healthy people, but according to Novikova [38], individuals of varying degrees of blindness display a correlation between the alpha reduction and the remaining visual acuity. The reports on reduced posterior alpha activity in the blind individuals correspond with other EEG studies that had found enhanced negative slow waves over the occipital brain of blind people [39,40]. Moreover, a reduction of the alpha band activity at a right-central electrode for blind compared to sighted adults has been reported during sleep by Bértolo [41]. Reduced visual acuity and lack of VEP response in the occipital cortex is one of the most commonly observed clinical symptoms in LHON [16]; hence, the observed reduction of the current source density in that area could be a consequence of the gradual loss of sight in that group, causing disconnection of the occipital cortex from the visual information flow. Nevertheless, we have also observed a correlation between the reduction of right hemisphere BA 19 source signal in the alpha frequency band and the average thickness of the right RNFL in the LHON participants, which suggest that there is a link between the changes in the retinal nerve fiber layer thickness and the reduction of EEG activity in the occipital cortex. Another study showed a unique process from thickening to thinning of the RNFL [42] and also that the optic tract fractional anisotropy value was significantly correlated with the thickness of the RNFL [7] in LHON participants. A comparison of previous findings and our results suggests that decreased alpha activity could progress faster with the degeneration of RNFL, but to confirm this thesis, a longitudinal study should be conducted.

The reduction of the EEG activity of the gamma frequency band in widespread areas was the second significant difference between the HC and LHON participants. The area of highest underactivity in the gamma band was placed bilaterally in the superior parietal lobule, right inferior parietal lobule, angular gyrus, and the right precuneus; however, decreased gamma activity was also observed in different cortical areas. The generalized weakening of resting-state neurophysiological activity in the gamma band is a frequently observed phenomenon in various diseases [43,44]. Nevertheless, at the level of the cortical microcircuit, gamma oscillations are generated through an interplay between excitatory (glutamate releasing) principal neurons and cortical inhibitors (gamma-aminobutyric-acid releasing) interneurons [45,46]. In vivo studies have demonstrated that inhibitor interneurons recruit pyramidal cells via inhibitors post-synaptic potentials [47]; hence, the local field potential of gamma oscillations that underlie the EEG signal are dependent on high rates of interneuronal spiking, as well as the population inhibitors’ synaptic activity. Kann et al. [48] showed the relationship between gamma activity abnormalities, mitochondrial gene expression, and oxidative metabolism in the hippocampus. Given that the main cause of LHON is a mutation in the mitochondrial DNA, one can assume that this disease etiopathogenesis may underlie gamma disturbances in such a large area of the brain. Nevertheless, to confirm these assumptions, a study that combines an mtDNA examination and the EEG should be conducted.

Application of a current source density reconstruction to the EEG signal analysis from the LHON and HC participants were found to be well matched and provided new information about neurophysiological changes in the case of the LHON patients. The usage of signal analysis frameworks or different methods allowed researchers to eliminate multiple errors from the EEG signal analysis [49]. In our study, the application of an algorithm based on a source density reconstruction allowed for estimating the distribution of neuronal activity sources that underlie the physiological changes in the brain. Additionally, we also avoided the volume conduction error from the analysis, which is very important in studies based on high-density EEG recordings. Nevertheless, in comparison to previous studies, we observed decreased neurophysiological activity in multiple cortical regions, which could have been impossible to observe with the classical analysis of EEG recordings.

This study has limitations that should be considered when interpreting its findings. First, there was no automated EEG artifact elimination (i.e., an independent component analysis (ICA)). Nevertheless, the selected signals were thoroughly evaluated by a clinical neurophysiologist with 40 years of experience; hence, bad epochs were excluded from the analysis. Second, the sample size was relatively small (*n* = 20), which might at least partially reduce the quality of the main findings. However, the disease is very rare in the Polish population; hence, the number of participants in our LHON sample was not typical for a neuroimaging study due to the specificity of rare diseases. The third limitation of our study is that we did not conduct an additional examination of the ophthalmic parameters of our patients since according to information taken from them in the initial interview, patients described themselves as functionally blind. Additionally, we correlated only the average RNFL results; hence, future studies should be extended to include an analysis of individual segments of the RNFL. Future studies should include younger relatives of patients, who are also burdened with the genetic risk of disease, but without clinical symptoms and blindness, to distinguish which neurophysiological alterations are directly connected with prodromal mitochondrial dysfunctions and which have functional consequences related to the advanced disease. Moreover, future studies should also analyze how therapy with idebenone could potentially change neurophysiological brain functions, such as neuronal activity.

## 5. Conclusions

In conclusion, this is the first resting-state EEG study that investigated changes in the whole-brain current source density of LHON participants. A comparison between controls and LHON patients revealed decreased neurophysiological activity in the alpha and gamma frequencies regarding various cortical areas. Furthermore, a reduction of the right hemisphere BA 19 source signal in the alpha frequency band was significantly correlated with the averaged thickness of the right RNFL in the LHON participants. Despite establishing relatively original and clinically relevant results, future longitudinal studies are necessary to track the progression of the detected neurophysiological changes in Leber’s disease.

## Figures and Tables

**Table 1:** The demographical and clinical values for both selected groups.

Clinical/Demographical Data:	LHON (*N* = 20) M (SD)	HC (*N* = 20) M (SD)	*t* or *χ^2^*	*p*
Age (years)	34.8 (12.18)	32.35 (6.46)	0.79	0.43
Education (years)	15.2 (1.96)	15.8 (1.98)	−1.02	0.31
Sex (% male)	90	90	0.17	0.99
Mitochondrial mutation 11778G > A (%)	100			
Duration of illness (months)	145.2 (132.75)			
Left RNFL averaged thickness (µm)	62.11 (2.83)			
Right RNFL averaged thickness (µm)	61.77 (3.13)			

Note: LHON—Leber’s hereditary optic neuropathy group, HC—healthy control group, M—mean, SD—standard derivation, *χ*^2^—Chi-square distribution, RNFL—retinal nerve fiber layer, *t*—Student’s *t*-test.

**Table 2:** Quantitative results of a between-group comparison regarding the current source density values in the alpha and gamma frequency bands that differentiated the groups.

Frequency Band	Lobe	Area	LHON (*N* = 20) M	HC (*N* = 20) M	*t*	*p*
Alpha	Occipital	Right Secondary Visual Cortex (BA 19)	23.43	61.13	−3.59	0.001
		Right Cuneus	18.66	52.20	−3.01	0.005
		Left Cuneus	19.12	44.46	−2.61	0.01
		Right Middle Occipital Gyrus	21.21	77.12	−3.86	<0.001
		Left Middle Occipital Gyrus	34.11	71.43	−2.68	0.01
	Temporal	Right Middle Temporal Gyrus	29.15	62.11	−2.66	0.01
	Parietal	Right Precuneus	26.12	57.88	−2.53	0.03
Gamma	Parietal	Right Superior Parietal Lobule	0.021	0.075	−4.11	<0.001
		Right Inferior Parietal Lobule	0.018	0.071	−4.01	<0.001
		Right Angular Gyrus	0.022	0.067	−4.03	<0.001
		Left Precuneus	0.011	0.034	−2.97	0.008
		Right Precuneus	0.013	0.049	−4.10	<0.001
	Occipital	Right Cuneus	0.031	0.048	−2.48	0.04
		Right Middle Occipital Gyrus	0.014	0.041	−2.77	0.01
		Right Superior Occipital Gyrus	0.017	0.053	−2.92	0.007
	Temporal	Right Middle Temporal Gyrus	0.033	0.073	−2.87	0.008
	Frontal	Right Superior Frontal Gyrus	0.044	0.065	−2.34	0.05
		Right Inferior Frontal Gyrus	0.039	0.068	−2.41	0.05

***Note***. M—mean; t—Student’s *t*-test.
